# The Role of Gut and Oral Microbiota in the Formation and Rupture of Intracranial Aneurysms: A Literature Review

**DOI:** 10.3390/ijms25010048

**Published:** 2023-12-19

**Authors:** Ann-Kathrin Joerger, Carolin Albrecht, Veit Rothhammer, Klaus Neuhaus, Arthur Wagner, Bernhard Meyer, Maria Wostrack

**Affiliations:** 1 Department of Neurosurgery, Klinikum Rechts der Isar, Technical University, 81675 Munich, Germany; annkathrin.joerger@tum.de (A.-K.J.); bernhard.meyer@tum.de (B.M.); 2 Department of Neurology, University Hospital Erlangen, Friedrich-Alexander University Erlangen Nuremberg, 91054 Erlangen, Germany; veit.rothhammer@fau.de; 3 Core Facility Microbiom, ZIEL Institute for Food & Health, Technical University of Munich, 85354 Freising, Germany; neuhaus@tum.de

**Keywords:** oral microbiome, gut microbiome, bacteria, periodontitis, intracranial aneurysm

## Abstract

In recent years, there has been a growing interest in the role of the microbiome in cardiovascular and cerebrovascular diseases. Emerging research highlights the potential role of the microbiome in intracranial aneurysm (IA) formation and rupture, particularly in relation to inflammation. In this review, we aim to explore the existing literature regarding the influence of the gut and oral microbiome on IA formation and rupture. In the first section, we provide background information, elucidating the connection between inflammation and aneurysm formation and presenting potential mechanisms of gut–brain interaction. Additionally, we explain the methods for microbiome analysis. The second section reviews existing studies that investigate the relationship between the gut and oral microbiome and IAs. We conclude with a prospective overview, highlighting the extent to which the microbiome is already therapeutically utilized in other fields. Furthermore, we address the challenges associated with the context of IAs that still need to be overcome.

## 1. Introduction

Subarachnoid hemorrhage (SAH) resulting from the rupture of an intracranial aneurysm (IA) is a devastating type of stroke affecting around 6/100,000 patients worldwide annually [1], leading to high mortality and morbidity rates [2,3]. It harbors a case fatality rate of 50% [4]. Due to the young age of onset compared to ischemic stroke and intracerebral hemorrhage, SAH is a major contributor to the stroke-related loss of productive life years despite advancements in risk assessment, imaging techniques, and surgical and intensive care treatment [2,3]. Most SAH survivors suffer from persistent, disabling neurological deficits; even those who experience some degree of neurological recovery often face ongoing psychological and cognitive impairments. As a result, 46% of SAH survivors remain severely disabled in their activities of daily life and are unable to return to work, resulting in a considerable socioeconomic burden [3,5]. Approximately 3% of the population harbors an incidental IA, but only a minority will experience a rupture leading to aneurysmal SAH [6]. While various risk factors for IA rupture have been identified, including smoking, prior SAH, hypertension, hypercholesterolemia, age, gender, aneurysm location, aneurysm size, heart disease, and aspirin use [7,8,9,10,11,12], their respective individual impact is far from being fully investigated [13,14,15].

In recent years, there has been a growing interest in the role of the microbiome in cardiovascular and cerebrovascular diseases [16,17,18,19,20,21,22]. The “microbiome” encompasses all microorganisms residing in or on various parts of the human body, which includes bacteria, fungi, and viruses (and all of their genes). The gut microbiome is particularly susceptible to modulation by dietary habits, lifestyle, and environmental factors [23]. The role of diet as a risk factor for cardiovascular events has been shown before [24]. More recently, attention has turned to the intestinal microbiome’s role in this process, with certain dietary components, such as carnitine from red meat and phosphatidylcholine from egg yolk, being metabolized by gut bacteria into trimethylamine, eventually converted in the liver into trimethylamine n-oxide (TMAO), which has been established as a risk factor for atherosclerosis [25,26]. For aortic aneurysms there is growing evidence about a potential role of the gut microbiome in formation and rupture [21,27,28,29]. For example, it was shown that patients with *Campylobacter gracilis* or *Fusobacterium* in their gut microbiome had a significantly higher incidence of aortic aneurysm-related events [27].

Moreover, the hypothesis exists that the microbiome regulates intracranial processes like neuroinflammation, brain injury, autoimmunity, and neurogenesis via the activation of innate and adaptive immune cells [30]. Several studies have demonstrated the crucial pathophysiological role of inflammation in the formation and rupture of IAs [31,32]. By modulating vascular inflammation, microbiota may exert both beneficial and detrimental effects on the development and rupture of IAs. It is worth noting that a substantial microbiome also exists in the oral cavity which could play a role in IA formation and rupture. So far, only a few studies have investigated the correlation between the microbiome and IAs. In this narrative review, we aim to explore the existing literature regarding the influence of the gut and oral microbiome on IA formation and rupture.

## 2. Exploring Pathways: Inflammation and Cerebral Aneurysm Formation, Gut–Brain Interactions, and Microbiome Analysis

### 2.1. The Role of Inflammation in Cerebral Aneurysm Formation

Increasing evidence suggests that inflammation plays a pivotal role in the formation of IAs [33]. This process includes endothelial dysfunction, followed by an inflammatory response, the phenotype shift of smooth muscle cells (SMCs), the remodeling of the extracellular matrix, and ultimately, cell death and degradation of the vessel wall [34,35]. The initial cause of endothelial dysfunction and subsequent vascular remodeling is the result of wall shear stress [36]. It was shown that areas of high wall shear stress, such as the apex of an arterial bifurcation, are especially predisposed to aneurysm formation [37]. Mechanical shear stress upregulates the expression of pro-inflammatory mediators, such as the nuclear factor kappa-light-chain-enhancer of activated B-cells (NF-κB) [38], matrix metalloproteinases (MMPs) [39], interleukin-1β (IL-1β) [40], Ets-1, and monocyte chemoattractant protein-1 (MCP-1) [41] and downregulates the expression of anti-inflammatory mediators, such as nitric oxide (NO) [40] in endothelial cells. Pro-inflammatory mediators activate the inflammatory response, in which macrophages play a pivotal role [33]. Macrophages not only release pro-inflammatory cytokines that attract more inflammatory cells, but also secrete MMPs that break down the extracellular matrix of the arterial wall, causing additional damage by promoting the activation of other proteinases. For rats, it was shown that the presence of macrophages and their derived MMPs was closely linked to IA growth, and that the inhibition of these MMPs haltered the progression of IAs [42]. Similarly, Kanematsu et al. [32] found that mice depleted of macrophages had a significantly reduced risk of developing IAs. Moreover, inhibiting MCP-1, a chemokine that controls the infiltration of macrophages, prevented the development of IAs in mice [43].

Macrophages are not the sole cells participating in the inflammatory response within the IA wall. Frosen et al. [44] reported in their study, which compared 42 ruptured and 24 unruptured IAs using a histological analysis, that the infiltration of the vessel wall by both macrophages and T cells was associated with aneurysm rupture. Moreover, mast cells may also play a role in IA formation. In rats, an elevated presence of mast cells during IA formation was noted [45]. Additionally, the advancement of IA was effectively halted when a mast cell degranulation inhibitor was administered.

SMCs, primarily located in the media layer of vessels, are the primary cells responsible for producing the extracellular matrix in the vascular wall [33]. During the early stages of aneurysm formation, SMCs migrate from the media layer into the intima layer in response to endothelial injury and undergo proliferation, resulting in myointimal hyperplasia. As the process continues, SMCs undergo a phenotypic shift from a specialized phenotype focused on contraction to a dedifferentiated phenotype, which contributes to inflammation and the breakdown of the extracellular matrix by expressing pro-inflammatory mediators and MMPs [34]. Morphologically, these dedifferentiated SMCs no longer maintain their tightly compacted spindle-like arrangement, but instead separate from each other and take on a spider-like appearance within the aneurysm walls, leading to remodeling [46].

MMPs are observed to be produced by both macrophages [42] and SMCs [45] within the wall of the blood vessels or aneurysms. These MMPs play a role in breaking down the extracellular matrix of the arterial wall, leading to additional damage through the upregulation of other proteinases and angiogenic factors [47].

Given this crucial role of inflammation in the pathophysiology of IA formation, the gut and oral microbiome could also be involved in this process by modulating the inflammatory response.

### 2.2. Potential Mechanisms of Gut–Brain Interaction

In rats, after ischemic stroke, an intestinal dysregulation with a greater permeability of the gut-blood barrier has been shown [48]. Consecutively, lipopolysaccharide (LPS) from Gram-negative bacteria of the intestine is translocated to the systemic circulation [49], activating inflammatory processes. Following cerebral ischemia, the disruption of the blood-brain barrier permits the entry of LPS into the brain parenchyma. This, in turn, triggers the activation of Toll-like receptor 4 (TLR4) and the release of inflammatory cytokines, further intensifying the damage to the ischemic brain [50]. Not only after ischemic stroke, but also after intracerebral hemorrhage (ICH), intestinal permeability increased in mice [51]. Moreover, T cells and monocytes originating from intestinal Peyer’s patches accumulated in the intracerebral hematoma. The expression of pro-inflammatory markers like IL-1β, inducible nitric oxide synthase, and tumor necrosis factor α (TNF-α) was significantly elevated in the brain tissue, while this was reversed after fecal microbiota transplantation.

**Figure 1:**
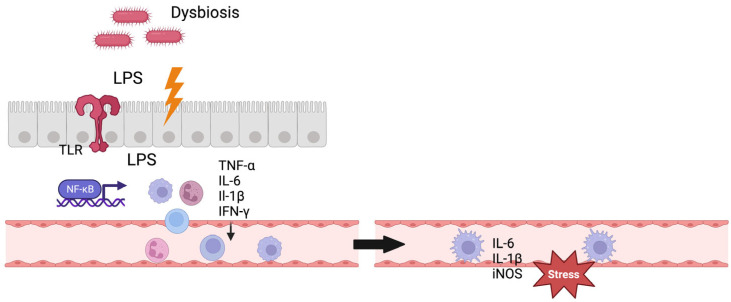
Gut–brain interaction. Figure shows a potential mechanism of gut–brain interaction. Gut dysbiosis leads to dysregulation of the gut–blood barrier and LPS translocation to systemic circulation, consecutively activating the immune system. The immune cells enter the intracranial vessels through the systemic circulation and exert stress on the vascular endothelium here through inflammatory mediators. TLR = Toll-like receptor, IL = interleukin, TNF = tumor necrosis factor, iNOS = inducible nitric oxide synthase, IFN = interferon. Created with BioRender.com.

For IA formation, the gut–brain interaction still remains unclear. A direct translocation of bacteria or LPS to the IAs appears unlikely [52]; instead, an indirect mechanism modulating the inflammatory response in the aneurysm wall is proposed [53] (Figure 1). Other potential mechanisms of gut–brain interaction include the direct stimulation of the enteric and autonomic nervous system, neuroendocrine pathways, and the production of biochemical (neuro-)transmitters by microbiota [49].

### 2.3. Methods of Analyzing the Microbiome

The two currently predominant approaches for microbial identification in microbiome samples involve the next-generation sequencing (NGS) of gene amplicons from marker genes, such as 16S rRNA, or shotgun metagenomics [54].

16S-rRNA gene amplicon sequencing: This approach is a targeted approach, i.e., with the help of the polymerase chain reaction (PCR), a marker gene of interest is amplified. The amplicons are then sequenced in high throughput and the sequences are used to identify an organism. The primary target for bacterial identification is normally the 16S-rRNA gene [54]. Due to its critical role in the ribosome, it is a well-conserved gene and suitable for the taxonomic classification of bacteria [55]. The 16S-rRNA gene sequence can be divided into invariable regions and nine variable regions (V1–V9). PCR is used with specific primers, which bind in the conserved regions. However, the most-used current sequencing machines only cover 2 × 300 bp, and therefore, only one to three (adjacent) variable regions are amplified. In the medical context, many 16S rRNA-based genotyping protocols focus on V1–V3, V3–V4, or the V4 regions, while, for instance, V5–V6 or V6–V8 are used more often in other fields (e.g., soil samples). Of note, despite their name, the invariable regions are also not completely fixed and primer bias occurs, since some primers may or may not bind certain taxa [56]. Novel long-read sequencers may cover the entire length of the 16S-rRNA gene, which can increase species-level resolution [57]. In any case, after sequencing, the data are used to identify and categorize microbial profiles for alpha and beta diversity and further advanced analyses (see [58] on Type 2 diabetes as example). Finally, a note of caution. Detecting bacteria, which may cause aneurysms but are only present in low numbers using an amplicon-based approach, is challenging [59,60]. For instance, the placenta microbiome has turned out to be purely due to contamination [61]. Future research must therefore use proper controls and care to avoid false conclusions.

Metagenome sequencing: This approach is an untargeted approach; i.e., where possible, the complete DNA of a given sample is isolated, fragmented, and sequenced (shotgun sequencing; [54]). Due to its untargeted nature, it could, in principle, detect all organisms present; however, this is limited by sequencing depth. For instance, biopsies might contain too-low numbers of bacteria and their DNA is “drowned” in human DNA. In contrast, in stool samples, where primarily only bacteria are found, one can uncover the genes, pathways, and metabolic functions existing within the community [62]. However, this still is limited, since the function of about 40–60% of the genes present in a given sample cannot be functionally predicted [63]. Nevertheless, deep-sequenced metagenome samples are certainly helpful in detecting bacteria, which might cause or have caused an aneurysm (see below).

## 3. The Gut Microbiome and Intracranial Aneurysm Formation and Rupture

While several studies have investigated the microbiome’s influence on stroke [17,18,19,64], there are limited data on the role of the microbiome in IA formation and rupture. The studies discussed here are depicted in Table 1.

**Table 1:** Overview of studies on the gut microbiome and IAs.

Study	Type	Medium	Intervention	Aim	Method	Result
Shikata et al., 2019 [52]	interventional study	mice	gut depletion by antibiotics in mice with IA induction vs. mice with normal gut and IA induction	- number and rupture rate of IAs; - number of macrophages in IA tissue; - mRNA levels of cytokines in IA tissue.	- immunohistochemistry; - RT-PCR.	- gut depletion reduced the incidence of IA (83% vs. 6%, *p* < 0.001) and rupture; - macrophage infiltration and mRNA levels of inflammatory cytokines were reduced with gut depletion.
Li et al., 2020 [65]	case–control study	- humans - mice	- analysis of fecal samples of 140 UIA and 140 control patients; - 20 mice treated with UIA patient feces and 20 treated with control feces.	- comparison of gut microbiome of patients with UIAs and without; - test, if changes in the gut microbiota influence the progression of UIAs in vivo.	- metagenomic shotgun sequencing; - serum metabolomic analysis.	- *Bacteroides* ssp., *Odoribacter splanchnicus*, *Clostridium* ssp. were significantly enriched in the UIAs; - *Hungatella hathewayi* was enriched in the control group; - microbiome of UIAs was significantly dominated by unsaturated fatty acid biosynthesis; - microbiome of controls was dominated by amino acid synthesis; - treatment with feces from UIA patients increased the overall incidence of IAs (85% vs. 45%; *p* = 0.019) and rupture rate (82% vs. 22%; *p* = 0.009); - serum concentrations of 2 of 8 fatty acids and 8 of 38 amino acids differed in mice transplanted with feces from UIA patients and controls.
Kawabata et al., 2022. [66]	multicenter, prospective case–control	humans	analysis of fecal samples of 28 RAs vs. 33 UIAs	comparison of gut microbiome of patients with UIAs and RAs	16S rRNA sequencing	- gut microbiome profile of UIAs and RAs were significantly different; - *Campylobacter* ssp. and *Campylobacter ureolyticus* were significantly higher in the RA group.
He et al., 2023. [67]	two-sample Mendelian randomization study	humans	database analysis of gut microbiome of patients with IA, UIA, SAH	association between the gut microbiome and the risk of IA, UIA, and SAH	inverse variance weighting approach	- Candidatus *Soleaferrea* decreased the risk of IA; - *Holdemania* and *Olsenella* increased risk of IA; - Lentisphaeria, Porphyromonadaceae, *Bilophila*, *Fusicatenibacter*, *Ruminococcus* sp. 1, Victivallales decreased risk of SAH; - Streptococcaceae increased risk of SAH; - Porphyromonadaceae, *Bilophila* decreased the risk of UIA; - Oxalobacteraceae, *Adlercreutzia*, *Intestinimonas*, *Victivallis* increased the risk of UIA.
Ma et al., 2023. [68]	two-sample Mendelian randomization study	humans	database analysis of gut microbiome of UIA patients	association between the gut microbiome and the risk of UIA	inverse variance weighting approach	- *Clostridia*, Rhodospirillaceae, *Adlercreutzia*, *Sutterella*, *Victivallis*, *Streptococcus*, Peptostreptococcaceae increased risk of UIA; - *Oscillospira*, *Paraprevotella* decreased the risk of UIA.

IA = intracranial aneurysm, UIA = unruptured intracranial aneurysm, RA = ruptured intracranial aneurysm, SAH = subarachnoid hemorrhage, RT-PCR = real time polymerase chain reaction, sp. = species (sg.); ssp. = species (pl.).

Kawabata et al. [66] observed significant differences in the gut microbiome between patients with unruptured intracranial aneurysms (UIAs) and those with ruptured intracranial aneurysms (RAs). The relative abundance of *Campylobacter*, especially *Campylobacter ureolyticus*, was larger in the RA group compared to the UIA group [66]. Nevertheless, it is well-established that brain injuries, such as ischemic stroke and ICH, can already have an impact on the gut microbiome [69], which raises uncertainty about whether the distinctions between the groups were influenced by the stress associated with SAH or had already manifested prior to the onset of SAH. A cause–effect relationship between *Campylobacter* and aneurysm rupture could not be shown.

In a database analysis of the gut microbiome of patients with UIAs and Ras, He et al. [67] identified three bacterial traits causally related to IAs and six bacterial traits related to UIAs (Table 1). Six bacterial traits were causally related to a decreased risk of subarachnoid hemorrhage (SAH) (Table 1).

Another Mendelian randomization study by Ma et al. [68] indicated bacteria with beneficial and detrimental effects on IAs as well (Table 1). However, both studies did not reflect other individual risk factors.

Nevertheless, these three studies reveal significant differences in the composition of the microbiome between patients with unruptured and ruptured IAs. They can even identify specific protective or non-protective bacterial species, suggesting that certain microbiome compositions could potentially serve as biomarkers for the risk of an aneurysm rupture in the future. However, correlation does not automatically imply causation. To identify biomarkers in the microbiome for the multifactorial event of a ruptured IA, it is necessary to explore the causes of the observed changes in the microbiome, to understand the mechanisms of signaling and interaction of the microbiome and the host, and to take into account ethnic variations in the composition of the microbiome [70] and other patient-specific risk factors.

Li et al. [65] observed that patients with UIAs had significant differences in their microbiome composition compared to healthy patients. They also found differences in metabolic pathways in the microbiome and differences in circulating amino acids between UIA patients and healthy controls. Moreover, transplanting feces from UIA patients into mice increased the incidence and rupture rate of IAs compared to mice treated with control feces, while supplementation with taurine significantly reduced the aneurysm formation and rupture rate. This study strongly indicates a role of the microbiome and of taurine on the formation and rupture of IAs. However, the exact mechanism could not be identified. Moreover, results from mice models should be transferred to the more complex human context extremely carefully.

Shikata et al. [52] demonstrated that gut depletion by antibiotics significantly reduced the incidence and rupture rate of IAs in mice, accompanied by a decrease in macrophages within the aneurysm wall and reduced levels of IL-1β, IL-6, and inducible nitric oxide synthase. Undoubtedly, this study indicates an influence of the microbiome on the development of IAs and the associated inflammatory response. However, the following aspects should be discussed: (I) The authors themselves acknowledge that they cannot explain the exact mechanism through which the microbiome affects IA formation. They could only rule out a direct migration of the bacteria into the cerebral arteries, as they did not find any bacterial DNA in the vessels. (II) The gut microbiota was eliminated by a combination of four antibiotics. However, these drugs themselves could have an influence on IA formation and the observed inflammatory response. (III) Antibiotic application not only depletes the gut microbiome but also microbiota in other sites, which also could contribute to the observed effects. (IV) Results from mice models are generated in a highly controlled setting that should be kept in mind when extrapolating the results to patients in a much more complex setting.

## 4. The Oral Microbiome and Intracranial Aneurysm Formation and Rupture

The literature on the role of the oral microbiome in intracranial aneurysm formation is limited. Table 2 gives an overview.

**Table 2:** Overview of studies on the oral microbiome and IAs.

Study	Type	Medium	Intervention	Aim	Method	Result
Pyysalo et al., 2013. [71]	prospective cohort study	humans	analysis of RA tissue of 36 patients with SAH	assess the presence of oral and pharyngeal bacterial genome in RAs	qRT-PCR	- bacterial DNA was detected in 21/36 (58%); - DNA from endodontic bacteria was detected in 20/36 (56%) and from periodontal bacteria in 17/36 (47%); - DNA of the Streptococcus-mitis group was the most common.
Pyysalo et al., 2016. [72]	prospective cohort study	humans	analysis of RA tissue of 42 patients and UIA tissue of 28 patients, tissue from healthy vessels and cardiac by-pass operations as controls	assess the presence of oral and pharyngeal bacterial DNA in RAs and UIAs	qRT-PCR	- bacterial DNA was detected in 49/70 (70%); - 29/42 (69%) of the RA tissue and 20/28 (71%) of the UIA tissue contained bacterial DNA of oral origin; - RA and UIA samples contained significantly more bacterial DNA than control samples.
Pyysalo et al., 2018. [73]	prospective cohort study	humans	analysis of tissue from gingival pockets of 30 patients with RA and 60 with UIA	assess the presence of dental infectious foci and odontogenic bacteria in patients before surgical treatment of IA	qRT-PCR	- total of 43% had gingival pockets of 6 mm or deeper; - bacterial and *Fusobacterium nucleatum* DNA were significantly higher in the patients with ≥6 mm gingival pockets than patients without them.
Inenaga et al., 2018. [74]	prospective cohort study	humans	analysis of saliva from 48 patients with CES, 151 with non-CES infarct, 54 with ICH, 43 with RA, and 97 with UIA vs. 79 healthy controls	assess the rate of Streptococcus mutans with collagen-binding protein, Cnm, in CES, non-CES infarct, ICH, RA, and UIA	PCR	- significantly high Cnm-positive rate was observed in CES, non-CES infarct, ICH and RA compared to controls.
Aboukais et al., 2019. [75]	prospective cohort study	humans	analysis of IA tissue from 10 patients with RA and 20 with UIA, samples from STA, dura mater, and MCA as control	assess the presence of bacteria in the walls of UIAs and RAs	PCR	- no bacterial presence was found in the wall of aneurysms.
Hallikainen et al., 2019. [76]	case series, case–control, prospective study	humans	oral examination of 42 patients with UIAs and 34 RAs compared to 5170 from prospective database	association of periodontitis with IA formation and SAH	multivariate logistic regression	- periodontitis, severe periodontitis, and gingival bleeding increased the risk of IAs significantly; severe periodontitis in ≥3 teeth or gingival bleeding increased the risk of SAH significantly.
Hallikainen et al., 2021. [77]	prospective cohort study	humans	analysis of serum of 227 IA patients, compared to 1096 from prospective database	association of IgA and IgG against *Porphyromonas gingivalis* and *Aggregatibacter actinomycetemcomitans* with IA and SAH	ELISA	- high IgA against *P. gingivalis* and *A. actinomycetemcomitans* increased the risk of IA and SAH significantly; - high IgG levels against *P. gingivalis* and *A. actinomycetemcomitans* decreased the risk of IA and SAH significantly.
Hallikainen et al., 2023. [78]	case–control, prospective study	humans	oral examination of 60 patients with UIA and 30 with RA compared to 5144 from prospective database	association of caries with IA formation and SAH	multivariate logistic regression	- caries does not increase the risk of IAs and SAH.

RA = ruptured intracranial aneurysm, UIA = unruptured intracranial aneurysm, SAH = subarachnoid hemorrhage, ICH = intracerebral hemorrhage, CES = cardioembolic stroke, qRT-PCR = real time quantitative polymerase chain reaction, STA = superficial temporal artery, MCA = middle meningeal artery, sp. = species (sg.), ssp. = species (pl.).

Pyysalo et al. [73] performed a dental examination of 89 patients before elective surgery for an IA. They detected gingiva pockets ≥6 mm as dental infection foci in 43% of patients. Moreover, total bacterial and *Fusobacterium nucleatum* DNA was significantly higher in patients with ≥6 mm gingival pockets than in patients without them. Nonetheless, it is important to note that these data do not permit us to draw any conclusion regarding a causal link between this observation and the formation of IAs.

In another study, Pyysalo et al. [71] detected DNA from endodontic and periodontal bacteria in 56% (20/36) and 47% (17/36) of tissue samples from ruptured IAs, respectively. The most frequently identified DNA belonged to the *Streptococcus mitis* group. Another study by Pyysalo et al. [72] found oral bacterial DNA in 69% (29/42) of ruptured and in 71% (20/28) of unruptured IA samples. Both tissue types contained significantly more bacterial DNA than control samples from non-atherosclerotic vessels walls. While these findings suggest a potential association between oral pathogens and IA formation, Aboukais et al. [75] could not detect bacterial DNA in any sample from ten ruptured and 20 unruptured IAs. A recently published review by Kennedy et al. [79] warns against interpreting studies with bacterial detection from low-biomass tissue. Their analysis of studies on the presence of bacteria in intrauterine prenatal tissue revealed that this is most likely contamination. Similarly, in the case of brain tissue, which is regarded as low-to-zero-biomass tissue, there is a high risk of results being distorted by contamination.

Hallikainen et al. [76] found that periodontitis was significantly associated with IAs and significantly increased the risk of SAH, while caries did not [78]. The association of periodontitis with the risk of IA formation and SAH was independent of gender, smoking status, hypertension, or alcohol abuse. The authors suggest the following mechanism: As periodontitis can accelerate the activation and mobilization of circulating neutrophils or monocytes, resulting in a generalized inflammatory response, it has the potential to influence the progression of cerebral artery remodeling and aneurysm pathology. This influence may render the artery more susceptible to aneurysm development and rupture. The lack of an association of caries with IAs and SAH could be explained by the fact that caries, contrary to periodontitis, does not predispose to bacteremia. In the case of periodontitis, there is a vulnerable surface of the gingiva that serves as an entry point for bacteria into the systemic circulation.

Another study by Hallikainen et al. [77] detected that serum IgA antibody levels against the two key periodontal pathogens, *Porphyromonas gingivalis* and *Aggregatibacter actinomycetemcomitans*, were significantly higher in patients with IAs compared to control patients. In a multivariate analysis, high IgA serum antibody levels against *P. gingivalis* and *A. actinomycetemcomitans* were significantly associated with a higher risk of IA formation and rupture, while IgG serum antibody levels against the same pathogens were significantly associated with a lower risk. Regarding this discrepancy, the authors provide the following rationale [77]: IgA levels predominantly signify recent or recurrent encounters with *P. gingivalis* and *A. actinomycetemcomitans*, whereas IgG levels are indicative of the development or triggering of an acquired immune response to these pathogens. Reduced IgG levels observed in IA patients may arise from several potential factors. One explanation is the capacity of *P. gingivalis* and *A. actinomycetemcomitans* to evade complement-mediated immune activation. Alternatively, it is conceivable that individuals may have developed immunity in response to prolonged pathogen exposure, without a concomitant increase in IgG levels. Furthermore, it is plausible that the quantity of circulating bacteria or bacterial metabolites/fragments may be insufficient to stimulate a significant elevation in IgG levels. Nonetheless, a limitation of this investigation lies in the exclusive measurement of IgA and IgG levels in serum, with no concurrent isolation of bacteria from the oral cavity. Furthermore, the study did not find any correlation between the clinical oral condition and the levels of serum antibodies.

Inenaga et al. [74] identified a significantly higher rate of *Streptococcus mutans* with collagen-binding protein, a bacterium with hemorrhagic characteristics, such as the activation of MMPs, in the saliva of patients with stroke, intracerebral hemorrhage, and ruptured intracranial aneurysm compared to a healthy control group. However, this was not a matched comparison, so the difference could be due to confounding factors.

## 5. Conclusions

The available evidence suggests that the gut and oral microbiome may play a role in the formation and rupture of IAs. Several studies have identified associations between oral and gut bacteria, periodontitis, gut microbiota depletion, unsaturated fatty acid biosynthesis, and IA pathophysiology. However, most studies are limited by a small sample size, the lack of matched controls, or are based on animal models, which hinder their ability to establish causality. The process of aneurysm formation in humans is complex and involves multiple factors, including genetics and exposure to risk factors. Animal aneurysm models are artificially generated and cannot reflect all these factors sufficiently.

## 6. Future Directions

In the field of chronic inflammatory bowel diseases and cancer, promising strategies have already emerged in the context of utilizing the microbiome [80,81]. Notably, fecal microbiota transplantation, which involves transferring fecal material containing distal gut microbiota from a healthy donor to a patient with an imbalanced gut microbiota, has been established as an effective therapy for recurrent *Clostridioides difficile* (former *Clostridium difficile*) colitis. Furthermore, the European Society of Clinical Microbiology and Infectious Diseases (ESCMID) has granted approval for the utilization of fecal microbiota transplantation in cases of recurrent diarrhea following antibiotic-associated diarrhea [80]. In the field of cancer research, there are numerous approaches to enhance the response to immunotherapy through the transplantation of various bacterial strains [81].

For IAs, further research is necessary to elucidate the exact mechanisms by which the gut and oral microbiome influence IA formation and rupture in humans. Prospective cohort studies and randomized controlled trials would provide higher-quality evidence for assessing these relationships. Moreover, the bias of contamination has to be addressed through a thorough experimental design. Understanding the role of the microbiome could potentially lead to new preventive strategies and therapeutic interventions for IAs.

## Data Availability

All data are provided in the text.
